# Acute neuromuscular fatigue, hormonal responses, and recovery in males during high-volume resistance exercises: Smith machine back squat vs horizontal leg press

**DOI:** 10.1007/s00421-025-05793-3

**Published:** 2025-05-05

**Authors:** Johanna Kotikangas, Simon Walker, Keijo Häkkinen, Heikki Peltonen

**Affiliations:** 1 Biology of Physical Activity, Faculty of Sport and Health Sciences, University of Jyväskylä, Jyvaskyla, Finland; 2 NeuroMuscular Research Center, Jyvaskyla, Finland; 3 School of Business, Sport Business by Jamk, Jamk University of Applied Sciences, Jyvaskyla, Finland

**Keywords:** Strength training, Neuromuscular performance, Endocrine system, Exercise selection, Exercise recovery, Peripheral fatigue

## Abstract

**Purpose:**

This study compared acute neuromuscular and hormonal responses and recovery in males during resistance exercises performed in Smith machine back squat (SQ) and horizontal leg press (LP).

**Methods:**

Twelve healthy, physically active men performed SQ and LP loadings consisting of 5 sets at ten-repetition maximum. Maximal bilateral isometric force and sEMG activity of quadriceps femoris over 500 ms (MIVC_EMG_) in isometric leg press, countermovement jump height, and resting twitch force were assessed before (PRE) and immediately after loadings (POST), and after recovery of 30 min (POST30), 24 h (POST24), and 48 h. Serum concentrations of cortisol (COR), growth hormone (GH), testosterone, and creatine kinase were assessed at the same time points and additionally after third set (MID). Blood lactate (BL) was measured at PRE, MID, POST, and POST30.

**Results:**

Total work performed was significantly higher during SQ than LP ( 43.0 ± 5.2 kJ vs. 29.1 ± 3.1 kJ, *p* < 0.001). All blood-based parameters increased significantly during both loadings (*p* < 0.01). COR and GH were significantly higher at MID, POST, and POST30, and BL at MID during SQ than LP (*p* < 0.05). Neuromuscular variables decreased significantly from PRE to POST during both loadings (*p* < 0.05), with an interaction observed in MIVC_EMG_ between SQ and LP (− 10.4 vs. 6.4%, *p* < 0.01). All variables had returned to baseline by POST24.

**Conclusion:**

SQ may provide a more potent stimulus for metabolic and hormonal responses during high-volume resistance exercise, at least partly due to the greater total work performed. Nevertheless, fatigue induced within the quadriceps femoris was similar between SQ and LP.

## Introduction

Smith machine back squat (SQ) and horizontal leg press (LP) exercises are commonly utilized to enhance force and power production in the lower extremities (Padulo et al. 2017; Migliaccio et al. 2018). Both exercises activate the same major lower-body muscle groups and involve comparable knee and ankle joint actions (Wilk et al. 1996; Escamilla et al. 2001; Padulo et al. 2017). However, the hip joint remains flexed throughout the LP exercises due to the seated position, whereas in SQ exercises, the hip joint extends fully at the end of the movement (Kang et al. 1996; Padulo et al. 2017). Due to this difference, the displacement of mass and total work per repetition is reduced during LP compared to SQ (Kang et al. 1996; Shaner et al. 2014; Padulo et al. 2017). Additionally, the Smith machine SQ is an axially loaded exercise in which the external load moves along a fixed vertical track (Schwanbeck et al. 2009; Migliaccio et al. 2018), whereas in the horizontal LP exercise, the sled moves along a horizontal track, minimizing the need to stabilize the trunk and lower-body joints (Kang et al. 1996; Clark et al. 2012; Wirth et al. 2016b; Fry et al. 2022).

Existing research indicates that free-weight SQ elicits greater muscle activation in quadriceps femoris and hamstring muscles compared to LP (Wilk et al. 1996; Escamilla et al. 1998; Escamilla et al. 2001) due to increased stabilization demands required for controlling SQ movement (Wilk et al. 1996; Escamilla et al. 1998; Escamilla et al. 2001). Moreover, the axial loading of SQ places greater demand on the trunk and antagonist muscles (Kang et al. 1996; Clark et al. 2012; Wirth et al. 2016b; Fry et al. 2022). Consequently, SQ may impose a greater neural demand on the lower-body muscles (Clark et al. 2012), although the external stability provided by the Smith machine reduces trunk and stabilizer muscle activation compared to free-weight SQ (Schwanbeck et al. 2009; Migliaccio et al. 2018). The Smith machine SQ also requires greater hip joint torque, especially at smaller knee angles, due to the increased hip flexion moment. Knee joint torque levels remain substantial throughout the Smith machine SQ and horizontal LP movements, but LP allows higher absolute force production due to reduced stabilization demands (Wirth et al. 2016a; Padulo et al. 2017).

Despite the aforementioned biomechanical differences, SQ and LP are often used interchangeably in resistance-training programs, particularly in recreational and rehabilitation settings aiming to develop quadriceps femoris hypertrophy and strength. However, the differences, for instance in muscle activation, joint torques, and overall workload, may result in distinct hormonal and neuromuscular fatigue responses (Kraemer and Ratamess 2005; Ahtiainen and Häkkinen 2009; Shaner et al. 2014; Migliaccio et al. 2018). For instance, significantly greater acute increases in serum concentrations of cortisol, growth hormone, and testosterone were observed after free-weight SQ loading consisting of six sets at a ten-repetition maximum (10RM) compared to inclined LP loading (Shaner et al. 2014). Another study observed significantly greater growth hormone response in inclined LP at 10RM, while free-weight SQ elicited greater responses at 25RM (Kang et al. 1996). These findings suggest that free weight SQ may elicit a more potent hormonal response compared to inclined LP. However, further research is needed to confirm whether similar responses occur with other loading exercises, such as Smith machine SQ and horizontal LP, differing in exercise execution and muscle activation, as machine-based exercise protocols may result in smaller acute increments in hormone concentrations (Schwanbeck et al. 2020; Fry et al. 2022). To the authors’ knowledge, no prior studies have directly compared acute neuromuscular fatigue responses between SQ and LP loadings within the same study. Nevertheless, several studies have reported substantial acute decreases in isometric force and dynamic power production following high-volume resistance exercise loadings, whether using Smith machine SQ (Pareja-Blanco et al. 2017; Kotikangas et al. 2022) or horizontal LP (Gorostiaga et al. 2012; Walker et al. 2015). While these studies provide insights into neuromuscular fatigue responses, direct comparisons are limited due to variations in loading protocols, including volume, intensity, and rest intervals (McCaulley et al. 2009; Walker et al. 2012; Peltonen et al. 2013; Corradi et al. 2021) in addition to exercise selection.

Given these gaps in the literature, research is needed to directly compare acute neuromuscular fatigue and hormonal responses between Smith machine SQ and horizontal LP during a high-volume resistance exercise protocol. By determining whether these two exercises provide similar training stimuli for neuromuscular and hormonal systems, the recommendation/practice of utilizing them interchangeably could be modified to optimize long-term resistance-training adaptations. In addition, the information generated by this research may advance the programming of resistance exercise by ensuring the effectiveness of training stimulus for neuromuscular and endocrine systems (Peltonen et al. 2018), thus enhancing the overall efficacy of resistance exercise (Hecksteden et al. 2015; He et al. 2018). Furthermore, recovery monitoring after resistance exercises aids in the evaluation of an individual’s readiness for the next training session and the periodization of training programs, since excessive fatigue could impair the formation of training adaptations (Pareja-Blanco et al. 2020). Thus, the aim of the present study was to examine and compare acute neuromuscular fatigue and hormonal responses during high-volume resistance exercise loadings using Smith machine SQ and horizontal LP exercise. Additionally, recovery of the neuromuscular and endocrine systems was monitored over 48 h. We hypothesized that SQ loading would demand greater muscle activation and total work performed, leading to greater acute neuromuscular fatigue and increases in serum hormone concentrations (Escamilla et al. 2001; Clark et al. 2012; Shaner et al. 2014). Furthermore, we expected SQ to result in a more prolonged recovery period compared to LP (Sousa et al. 2024).

## Methods

### Study protocol

The study was structured into a familiarization session, two separate high-volume resistance exercise loading sessions, and four recovery sessions. All participants performed back squat loading (SQ) using a Smith machine (Kraftwerk, Finland) during the first loading session and leg press loading (LP) in horizontal leg press (David F210, David Health Solutions Ltd, Finland) during the second loading session. The familiarization session and both loading sessions were separated at least by 1 week to minimize the potential impact of earlier loading on subsequent session. The order of the loading sessions was not randomized due to scheduling constraints within the larger research project from which the present data were collected.

Neuromuscular variables were assessed before (PRE) and immediately after the loadings (POST). Acute hormonal responses, along with blood lactate and glucose concentrations, were measured after the third loading set (MID) in addition to PRE and POST. The recovery of neuromuscular and endocrine systems was monitored after 30 min (POST30), 24 h (POST24), and 48 h (POST48) of recovery. All measurement sessions were conducted between noon and 9 PM, since neuromuscular performance generally peaks during the afternoon hours (Ayala et al. 2021) concurrently with the stabilization of the diurnal fluctuations in hormone concentrations (Hayes et al. 2010). In addition, each participant was tested at the same time of day (± 1.5 h) to minimize diurnal variations in neuromuscular performance and hormone concentrations (Hayes et al. 2010; Ayala et al. 2021).

### Participants

The present study involved 12 healthy, recreationally active men (28.0 ± 3.3 y, 179.3 ± 6.2 cm, 85.2 ± 6.2 kg). All participants regularly engaged in various recreational activities but did not follow a systematic resistance-training program (i.e., no more than one resistance exercise session per week). All participants had some prior experience with SQ and LP exercises, and they were able to perform both exercises using the correct technique. Potential candidates were carefully screened using a medical history questionnaire and a resting electrocardiogram reviewed by a cardiologist before participation. The potential candidates were excluded from the study if they had musculoskeletal, cardiovascular, endocrine, or neurological disorders, had sustained musculoskeletal injuries within 6 months before the study, or were using any medication or performance-enhancing substances that could affect the neuromuscular or endocrine systems. All participants were informed about the study design, test procedures, and the potential benefits and risks associated with the study before they signed the informed consent document. The study was approved by the Human Sciences Ethics Committee of the University of Jyväskylä (ethical approval number: 1369/13.00.04.00/2022) and was conducted in accordance with the Declaration of Helsinki (2013).

### Loading exercises and protocol

**Fig. 1:**
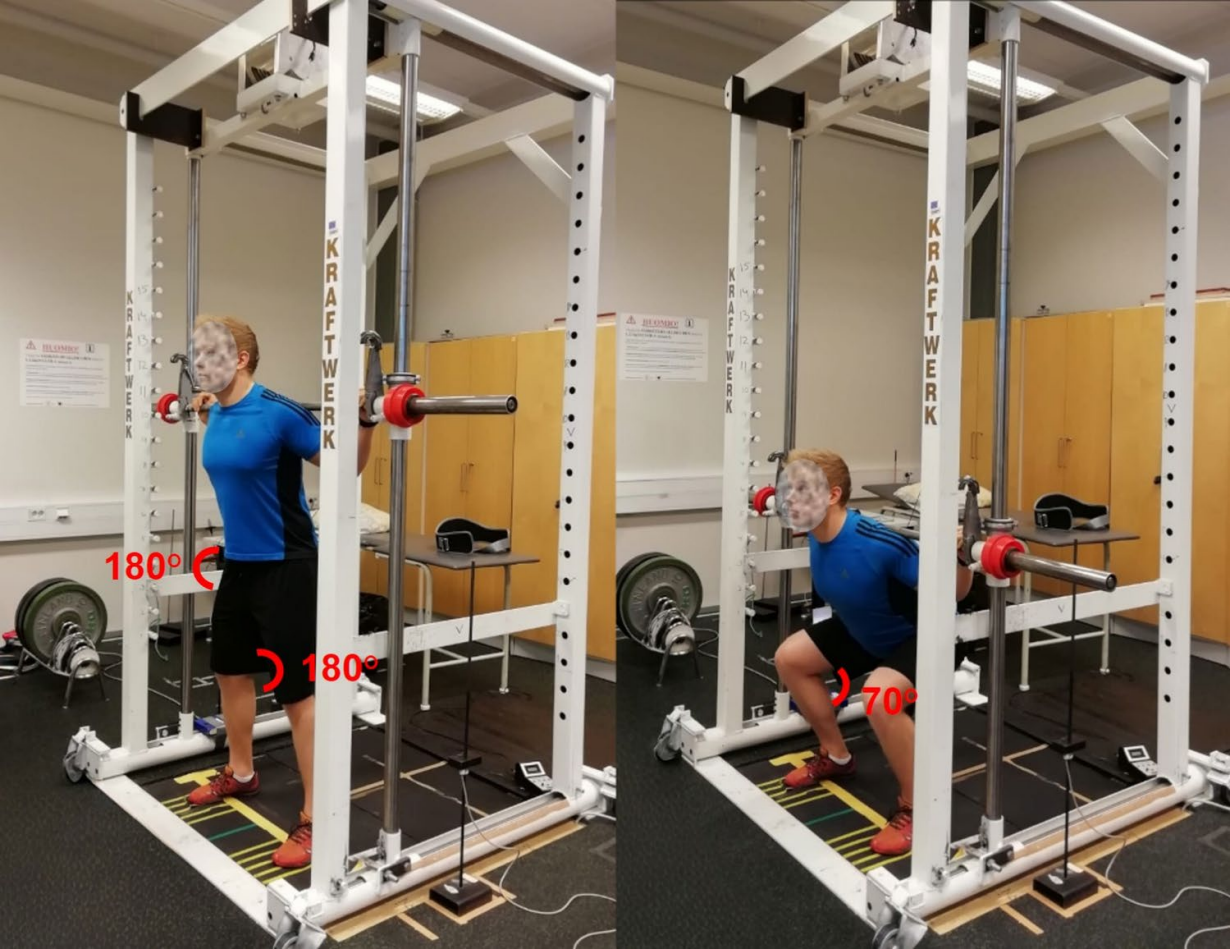
Back squat exercise performed in the Smith machine device

**Fig. 2:**
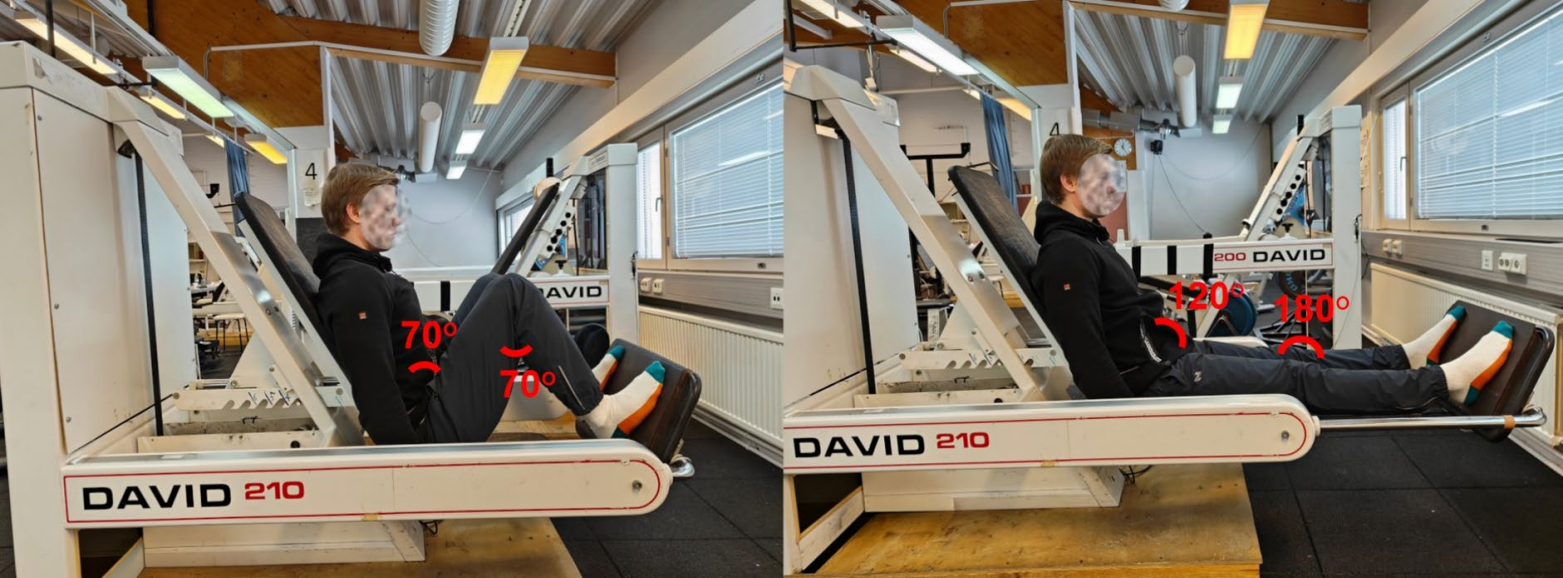
The leg press exercise performed in the horizontal leg press

The two loading exercises were SQ in a Smith machine (Kraftwerk, Finland) and LP in a horizontal leg press machine (David F210, David Health Solutions Ltd, Finland). In the Smith machine, the barbell was guided along a fixed vertical path between two parallel tracks. A reference line was drawn on the ground directly beneath the barbell, and participants were instructed to align the midfoot (arch) with this line. The participants were instructed to lower their body toward the ground in a controlled manner until they reached a 70° knee angle (180° = full extension) indicated by an audible signal from a photosensor. The hip angle at the bottom position of SQ varied between the participants due to anthropometric differences. After hearing the signal, the participants extended their lower limbs and trunk back to the starting position (180° hip and knee angle, Fig. 1). For LP, the participants started in a seated position with a knee and hip angle of 70°. They pressed the sled along a linear horizontal track until achieving full knee extension (180°) and a hip angle of 120°, and then flexed their lower limbs in a controlled manner back to the starting position (Fig. 2).

Both loadings consisted of five sets of ten repetitions with 1.5 min between-set rest intervals. The initial load was set at 70% of one-repetition maximum (1RM) for SQ and 90% of 1RM for LP as they were determined to represent approximately a ten-repetition maximum (10RM) based on pilot measurements. The loads of subsequent sets were adjusted to match the actual 10RM load based on the participants’ performance, which was closely monitored by the researchers. If participants successfully completed all repetitions unassisted, the load was maintained or increased; if assistance was required, the load was reduced. The participants performed all repetitions at a self-selected normal tempo and, if needed, the spotters provided assistance during the concentric phase of the last repetitions of sets.

### Familiarization session

The familiarization session began with a 10 min warm-up, which included cycling on a stationary bicycle and performing dynamic stretching exercises instructed by the researchers. Subsequently, all test devices were adjusted for the participants, and the settings of each device were recorded. For the bilateral isometric leg press dynamometer and the unilateral isometric knee extension device, a knee angle of 107° and a hip angle of 110° were determined using a hand-held goniometer. In the Smith rack, participants selected their stance width from marked positions on the ground at 5 cm intervals and familiarized themselves with the SQ movement. Next, the participants were asked to pause in the base position of SQ, where a knee angle of 70° was measured using a hand-held goniometer, and the height of the infrared beam of a custom-built photosensor (Faculty of Sport and Health Sciences, University of Jyväskylä, Finland) was adjusted to match this knee angle. In the LP machine, the same stance width as in the Smith machine was used, and the backrest position was adjusted to achieve a starting knee angle of 70°. After setting up the devices, instructions for each test procedure were given, and participants were able to familiarize themselves with all test procedures.

Given that participants were uncertain about their 1RM result, a three-repetition maximum (3RM) test in SQ was performed at the end of the familiarization session. A standardized warm-up included 4 min of cycling on a stationary bicycle, followed by warm-up sets consisting of 2 sets of 10 repetitions with a 16 kg barbell and 1 set of 6 repetitions with a 40 kg load. Thereafter, the participants performed sets of three repetitions with weight increments ranging from 2.5 to 25 kg between sets until the 3RM was achieved. The weight increments were based on participants’ personal experiences and their performance evaluated by the researchers. A 3 min rest period was provided between each set.

After 3RM test, the positions of the surface EMG (sEMG) electrodes for vastus lateralis (VL), rectus femoris (RF), and vastus medialis (VM) were marked with a permanent marker following SENIAM guidelines (Hermens et al. 2000). The participants were advised to reinforce these markings after sweating or showering to ensure that electrodes were placed in the same position during both loadings. At the end of the familiarization session, the participants were given specific preparation instructions for the loading sessions. They were instructed to abstain from additional exercise, supplements, and alcohol for 24 h before the loading sessions. They were also instructed to consume a meal 1.5–2 h before the loading sessions, limit their caffeine intake to a maximum of one cup of coffee per day, and ensure at least 8 h of sleep per night during the measurement weeks. However, the researchers did not systematically monitor participants’ dietary intake nor sleep patterns. During the loading sessions, the participants were provided with 0.5 deciliters of water, which they were required to consume.

### Neuromuscular assessments

#### One-repetition maximum test

At the beginning of both loading sessions, sEMG electrodes were placed on the muscles, and a fingertip blood sample was taken. After the standardized warm-up, three warm-up sets were performed with a 2 min rest interval: the first set consisted of four-to-five repetitions with a load of 65% of the estimated 1RM, the second set was three repetitions with a load of 80% of the estimated 1RM, and the third set was one repetition with a load of 90% of the estimated 1RM (i.e., the load determined during the 3RM test in the familiarization session). After the warm-up sets, the participants performed 1RM attempts with 3-min rest intervals and load increments of 1.25 to 10 kg in SQ and 2.5 to 20 kg in LP until failure. The 1RM result was achieved after two-to-five attempts. The estimated 1RM for SQ was based on the 3RM obtained during the familiarization session, and for LP, it was based on the 1RM obtained in SQ. The ratios between 1RM and body mass were calculated for each participant, allowing a comparison between SQ and LP exercises. The test–retest values from our laboratory for 1RM tests were an intraclass correlation coefficient (ICC) = 0.992 and a coefficient of variation percentage (CV%) = 2.8 for SQ, and ICC = 0.935 and CV% = 3.9 for LP.

#### Maximal isometric voluntary contraction in isometric leg press

Maximal bilateral isometric force (MIVC) was assessed using a custom-built isometric leg press dynamometer (Faculty of Sport and Health Sciences, University of Jyväskylä, Finland). The participants were instructed to exert force against the force plate as fast and hard as possible, maintaining a maximum effort of approximately 3–5 s. Strong verbal encouragement was provided during each trial to ensure maximal effort. Three trials with a 1-min rest interval between trials were performed at PRE, POST24, and POST48. At POST and POST30, three trials were performed with a brief 15–20 s rest interval. An additional trial was performed if force increased by over 5% during the final trial. MIVC was defined as the highest force value (newtons, N) recorded during the force–time analysis (Häkkinen et al. 1998, Fig. 3). The test–retest reliability for MIVC was ICC = 0.941 and CV% = 6.6.

**Fig. 3:**
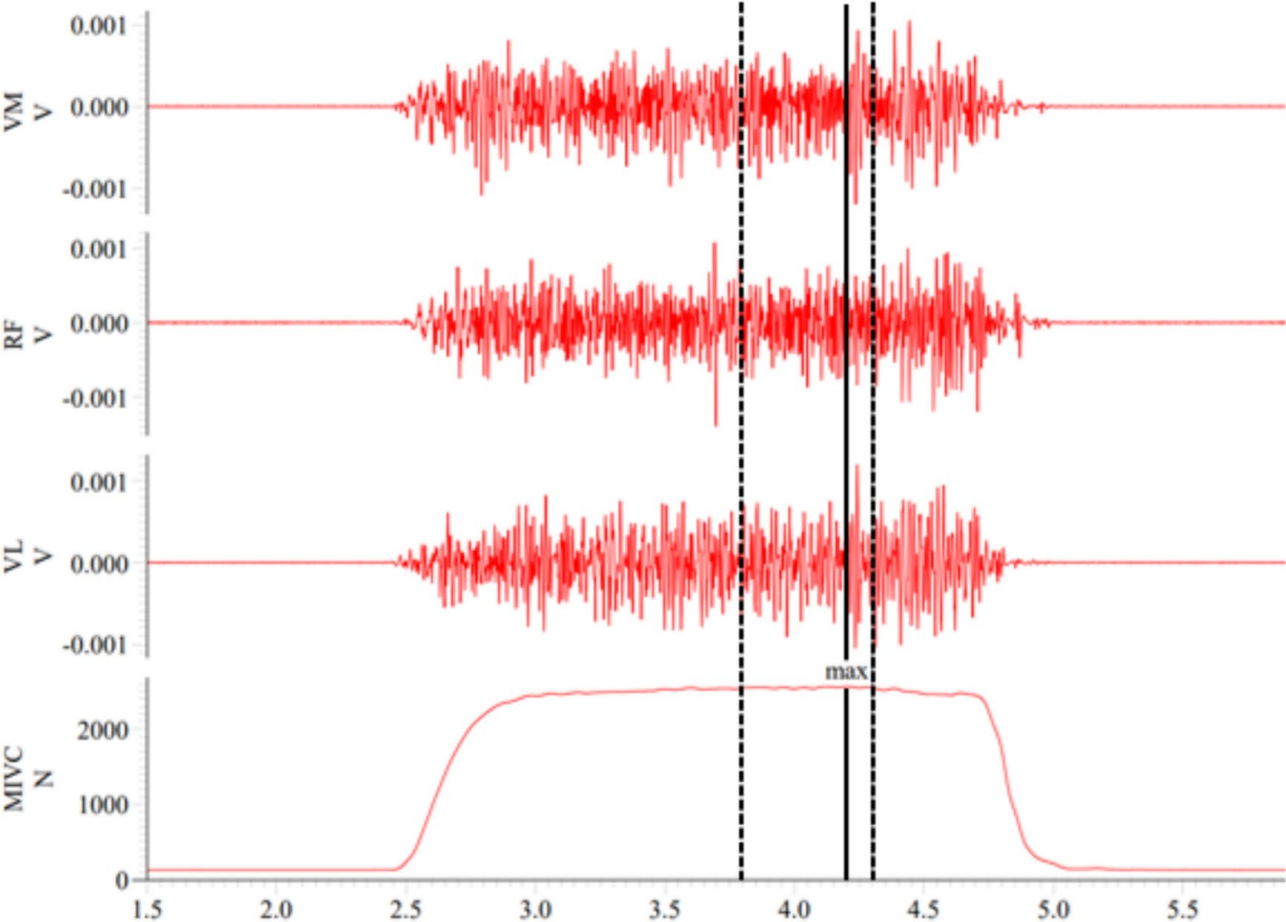
Representative isometric force and sEMG activity signals assessed during PRE measurements using an isometric leg press dynamometer. A black solid line indicates maximal bilateral isometric force (MIVC) measured during the trial, and black dashed lines show a 500-ms window for maximum force plateau where maximum sEMG amplitudes for vastus medialis (VM), rectus femoris (RF), and vastus lateralis (VL) were obtained

#### Surface electromyography (sEMG) in isometric leg press

Surface electromyographic activity (sEMG) of VL, VM, and RF muscles was recorded in isometric leg press using a Telemyo 2400R telemetric recording system (Noraxon, Arizona, USA). Silver-silver chloride surface electrodes (Ambu BlueSensor N, Copenhagen, Denmark) with dimensions of 30 × 22 mm, an inter-electrode distance of 20 mm, and an AC impedance of 600 Ω, were placed on the aforementioned muscles of the right lower limb following careful skin preparation. Raw sEMG signals were sampled at 2000 Hz and amplified (500 gain) with a bandwidth of 10–500 Hz. sEMG signal was transmitted to a receiver box and fed through an A/D converter (Micro 1401, Cambridge Electronic Design Ltd., Cambridge, United Kingdom) to a computer where Signal 4.14 software (Cambridge Electronic Design Ltd., Cambridge, United Kingdom) was used for data recording and subsequent offline analyses. The recorded sEMG data were filtered using a 20–350 Hz band-pass filter before further analyses. Maximum sEMG amplitudes were obtained from the root mean square over 500 ms during maximal force plateau. After the analyses, VL, VM, and RF amplitudes were averaged (VL + VM + RF/3) to represent the maximal sEMG of the quadriceps femoris muscle (MIVC_EMG_). The test–retest reliability for MIVC_EMG_ was ICC = 0.678 and CV% = 15.9.

#### Countermovement jump height

Countermovement jump height (CMJ) was measured using a contact mat (Faculty of Sport and Health Sciences, University of Jyväskylä, Finland). At the beginning of the trial, participants rapidly lowered their bodies toward the ground, then changed the direction of movement as fast as possible, and pushed off from the ground straight upward. The squat depth was self-selected, and participants were required to keep their hands on their hips throughout the trial. Participants performed two-to-three trials without any additional rest between the trials at all time points. Jump height was calculated using the flight time measured by the contact mat. The equation was h = gt^2^/8, where h = countermovement jump height, g = acceleration due to gravity, and t = flight time. The test–retest reliability for CMJ was ICC = 0.959 and CV% = 4.7.

#### Femoral nerve electrical stimulation

Femoral nerve electrical stimulation was performed in a custom-built isometric unilateral knee extension device (Faculty of Sport and Health Sciences, University of Jyväskylä, Finland). The participants were secured in a seated position over the pelvis and the distal part of the thigh using belts, and their right ankle was secured with a Velcro strap connected to a strain gauge-based force measurement module. The femoral nerve was located by palpating the femoral pulse from the femoral triangle, and two self-adhesive square electrodes with dimensions of 50 × 50 mm (Polar Trode, Espoo, Finland) were placed on either side of the femoral nerve. Double 1-ms rectangular pulses with a 10 ms inter-stimulus interval and increases of 20 mA were delivered to the relaxed muscle by a constant-current stimulator (Model DS7AH, Digitimer Ltd, United Kingdom) until the torque response plateaued. A 30% increase in stimulation intensity was added to ensure supramaximality of the stimulus. After the determination of stimulus intensity, two 3–5-s maximal voluntary contractions were performed without stimulation. The first supramaximal double-pulse electrical stimulus was delivered to the relaxed muscle after the third maximal contraction. In total, three stimulations with a 1-min rest interval between maximal voluntary contractions were given at PRE, POST24, and POST48, and two stimulations with a brief 15–20 s rest interval between maximal voluntary contractions were provided at POST and POST30. Potentiated isometric rest twitch force (RT) was defined as the highest force value (N) recorded. The test–retest reliability for RT was ICC = 0.901 and CV% = 5.0.

#### Average concentric power within loading sets

Average concentric power (AP) of each SQ and LP repetition was assessed within the loading sets using customized scripts. The equation used to calculate average concentric power was AP = F × (d/t), where AP = average concentric power (W), F = force (the weight of the external load, N), d = the displacement of the barbell or footplate (m), and t = time (s). Based on previous research (e.g., Pataky et al. 2003), it was estimated that approximately 88% of body mass was involved in SQ exercise (excluding the mass of the leg proximal to the knee). Thus, when calculating AP for SQ repetitions, both the weight of the external mass and 88% of body mass were included in the calculations. To determine percentage changes in AP within the loading sets, the values of individual repetitions were compared to the value of the second repetition of the first set (SET1REP2). The test–retest reliability for AP in SQ was ICC = 0.989 and CV% = 4.0.

#### Total work and total duration of sets

Total work during each loading set was calculated using the equation (external mass × 9.81 m*s^−2^) × vertical displacement × number of repetitions. In a similar manner to the determination of AP, it was estimated that approximately 88% of body mass was involved in SQ exercise excluding the mass of the shank and foot (e.g., Pataky et al. 2003). Thus, the equation utilized to calculate total work during SQ was [external mass + (0.88 × body mass)] × 9.81 m*s^−2^ × vertical displacement × number of repetitions. Due to the horizontal movement pattern of LP, body mass likely had minimal effect on the total work performed and thus was not taken into account. The total duration of sets was calculated by summing the durations of the eccentric and concentric phases of all individual repetitions, excluding any possible pauses between the repetitions.

### Venous blood samples

Venous blood samples were collected at PRE, MID, POST, POST30, POST24, and POST48 using sterile needles in serum tubes (Vacuette, Greiner Bio One International GmbH, Austria). The samples were allowed to clot at room temperature for approximately 30 min before being centrifuged for 15 min at 3600 RPM (Megafuge 1.0R, Heraeus, Germany). Following the separation of serum, the samples were initially stored at − 20 °C and then transferred to − 80 °C until analysis. Serum concentrations of testosterone (TES), cortisol (COR), and growth hormone (GH) were analyzed using the Immulite 2000 XPi Immunoassay System (Siemens Healthcare Diagnostics Inc., Tarrytown, NY, United States), and creatine kinase (CK) was analyzed using the Indiko Plus Clinical Chemistry Analyzer (Thermo Fisher Scientific, Vantaa, Finland). The sensitivities and intra-assay coefficient variances for serum hormones were TES 0.5 nmol/L and 8.2%, COR 5.5 nmol/L and 7.9%, GH 0.01 ng/mL and 5.8%, and CK 2.2 U/L and < 0.9%, respectively.

### Blood lactate and glucose concentrations

Capillary blood samples were taken from the fingertip at PRE, MID, POST, and POST30 to determine blood lactate (BL) and glucose (GLUC) concentrations. Each sample was collected into a 20 µL capillary tube and then mixed with a 1 mL hemolyzing solution. The samples were analyzed after the completion of the loadings using a Biosen lactate analyzer (S_line Lab + , EKF Diagnostic, Magdeburg, Germany).

### Statistical analyses

Statistical analyses were conducted using IBM SPSS Statistics version 28 (SPSS, Inc., Chicago, Illinois, USA). The normal distribution of the data was verified using the Shapiro–Wilk test. As GH and CK data were not normally distributed, they were transformed to a logarithmic scale (log_10_). The results are presented as group mean and individual values, standard deviations (SD), and percentage changes (%). Two-way (loading [2] × time point [5]) repeated-measures analysis of variance (ANOVAs) were used to examine differences in neuromuscular, TES, COR, and CK responses from PRE to POST48, and two-way (loading [2] × time [4]) ANOVAs in GH, BL, and GLUG responses from PRE to POST30 between loadings. In addition, three-way (loading [2] × set [3] × repetition [3]) ANOVAs were utilized to compare differences in AP between loadings within the sets. Pairwise comparisons were conducted using Bonferroni’s significance test, with Greenhouse–Geisser correction applied when necessary. The level of significance for all tests was set at *p* ≤ 0.05. Effect sizes for the pairwise comparisons were determined by Cohen’s d, where small = 0.2, medium = 0.5, and large = 0.8 effects were defined. Pearson’s product–moment coefficient was used to assess correlations between variables. The strengths for correlations were the following *r* = 0–0.19 very weak, *r* = 0.20–0.39 weak, *r* = 0.40–0.59 moderate, *r* = 0.60–0.79 strong, *r* = 0.80–1 very strong.

Individual responsiveness to the resistance loadings was defined as a change larger than the typical error of measurement (TE). TE was calculated as the standard error of within-participants standard deviation for PRE values of SQ and LP and this value was multiplied by 2 to express TE (Hopkins 2000). In addition, the smallest worthwhile change (SWC) was calculated for all variables by multiplying the between-subjects standard deviation for PRE values of SQ and LP by 0.2. TE and SWC are expressed as a percentage change (neuromuscular variables) or absolute change (hormonal and metabolic responses) of the conditions’ mean.

When designing the study, it was expected that 12 persons would be a sufficient sample size to find meaningful differences between the present loadings. This expectation was based on other similar studies comparing the effect of exercise selection on neuromuscular and/or hormonal responses (e.g., Escamilla et al. 2001; Peltonen et al. 2013; Shaner et al. 2014), which have commonly utilized a sample size of 10–15 persons. In these studies, significant differences in a broad range of neuromuscular and hormonal variables have been found between the loadings utilizing different exercise devices.

## Results

### Total work and duration of sets

Total work performed during SQ was significantly higher compared to LP (*p* < 0.001, Table 1). No significant difference in the total duration of sets was observed between the loadings, although there was considerable inter-individual variability.

**Table 1:** Mean (± SD) one-repetition maximum (1RM, kg), 1RM:body mass ratio, set loads (kg), external and system masses (kg), vertical displacement (m), total work (external mass × 9.81 ms^−2^ × vertical displacement × number of repetitions)^#^, and total duration of sets (s) during smith back squat and horizontal leg press loadings

Loading	Smith back squat	Horizontal leg press
1RM (kg)	116.1 ± 14.2^***^	227.7 ± 25.6
1RM/body mass	1.37 ± 0.18^***^	2.69 ± 0.35

	External mass / System mass (kg)		External mass / System mass (kg)
SET1	81.8 ± 10.3^***^ / 156.8 ± 12.6^***^		200.9 ± 31.7
SET2	81.6 ± 10.5^***^ / 156.5 ± 12.5 ^***^		199.1 ± 28.4
SET3	78.0 ± 9.6^***^ / 153.0 ± 10.8^***^		197.6 ± 27.1
SET4	73.0 ± 9.4^***^ / 147.9 ± 10.2^***^		195.6 ± 27.3
SET5	67.8 ± 13.0^***^ / 142.7 ± 13.1^***^		195.6 ± 27.1
Vertical displacement (m)	0.58 ± 0.06^***^		0.31 ± 0.03
Total work (kJ)	43.0 ± 5.2^***^		29.1 ± 3.1
Total duration of sets (s) (*n* = 11)	134 ± 13		131 ± 27

^***p<0.001 refers to significantly different from horizontal leg press^

^#During Smith back squat loading, total work was calculated using system mass, i.e., (external mass+ (body mass×0.88)) ×9.81 ms−2×vertical displacement×number of repetitions)^

### Effect of total work on blood-based parameters

**Fig. 4:**
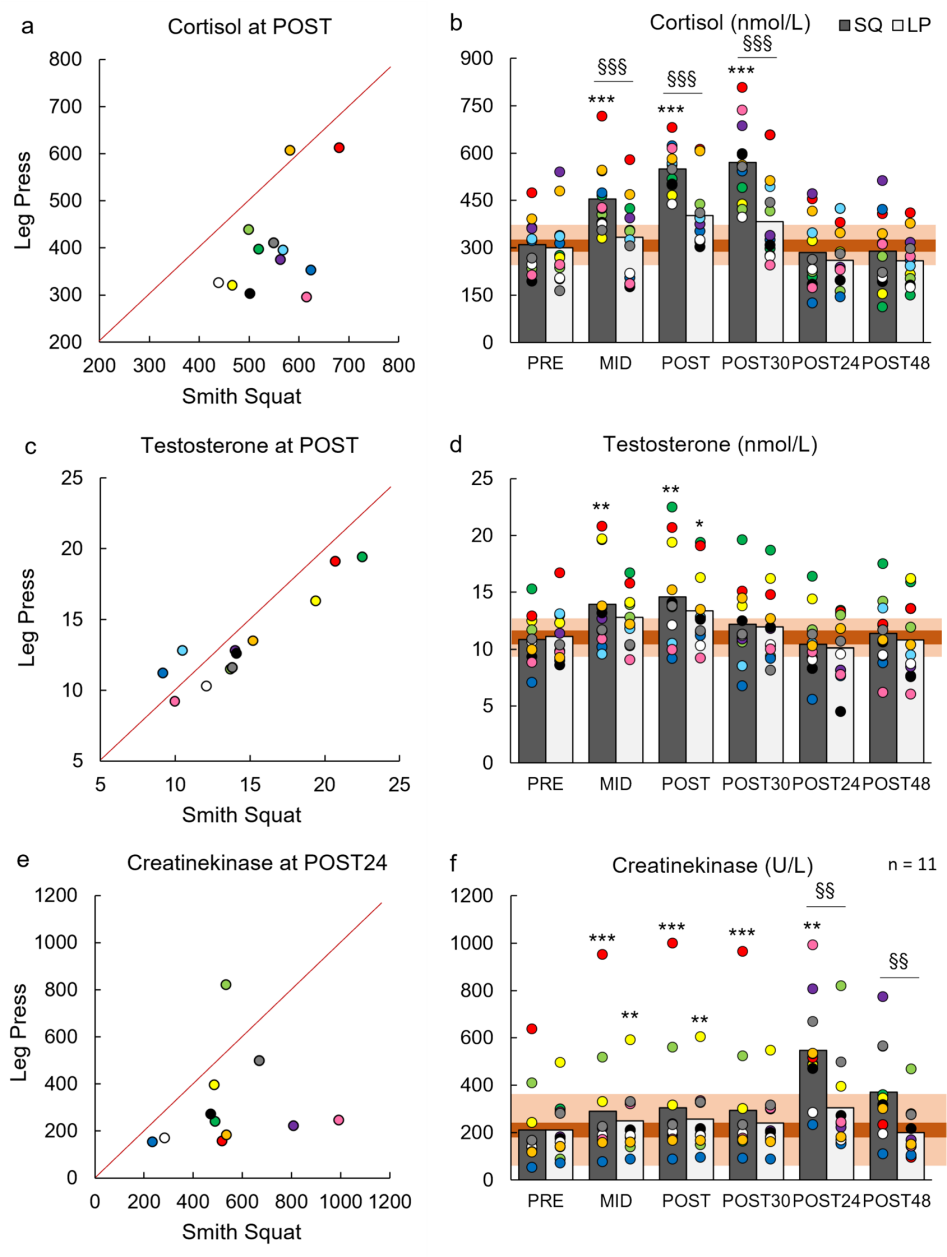
Comparison of serum concentrations of cortisol (nmol/L, a), and testosterone (nmol/L, c) immediately after (POST) Smith back squat (SQ) and horizontal leg press (LP) loadings and of creatine kinase (U/L) after 24-h recovery (POST24). In addition, mean and individual serum concentrations of cortisol (nmol/L, b), testosterone (nmol/L, d), and creatine kinase (U/L, f) measured before the loadings (PRE), after the third set (MID), POST, and after recovery of 30 min (POST30), 24 h (POST24), and 48 h (POST48). The light orange-shaded area represents responsiveness to loadings (i.e., change larger than two times the typical error of measurement) and the dark orange-shaded area represents the smallest worthwhile change thresholds (0.2 × between-participants standard deviation). *p < 0.05, **p < 0.01 and ***p < 0.001 significantly different from PRE; §*p* < 0.05, §§*p* < 0.01 and §§§*p* < 0.001 significantly different from LP

**Fig. 5:**
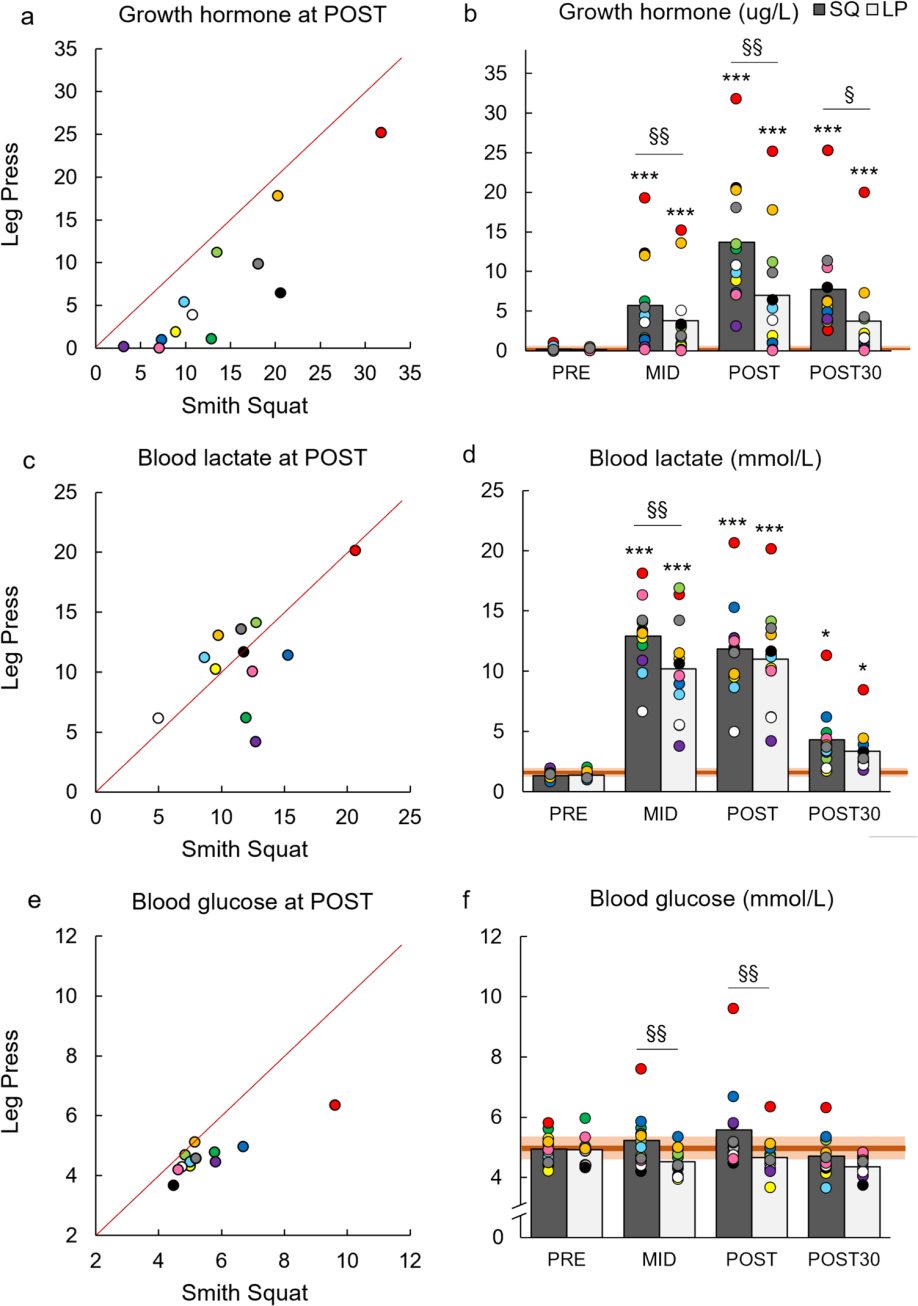
Comparison of serum concentration of growth hormone (ug/L, **a**), lactate (mmol/L, **c**), and glucose (mmol/L, **e**) immediately after (POST) Smith back squat (SQ) and horizontal leg press (LP) loadings. In addition, mean and individual serum concentrations of growth hormone (ug/L, **b**), lactate (mmol/l, **d**), and glucose (mmol/l, **f**) measured before the loadings (PRE), after the third set (MID), POST, and after recovery of 30 min (POST30). The light orange-shaded area represents responsiveness to loadings (i.e., change larger than two times the typical error of measurement) and the dark orange-shaded area represents the smallest worthwhile change thresholds (0.2 × between-participants standard deviation) *p < 0.05, and ***p < 0.001 significantly different from PRE; §*p* < 0.05, and §§*p* < 0.01 significantly different from LP

Significant loading × time interactions were found for COR, GH, BL, and GLUC (Table 2). Pairwise comparisons indicated that COR and GH were significantly higher in MID, POST, and POST30 (*p* < 0.05), BL in MID (*p* < 0.01), and GLUC in MID and POST (*p* < 0.01) during SQ compared to LP (Figs. 4 and 5). Additionally, significant effects for time were observed in the concentrations of all hormones, CK, BL, and GLUC. Total work correlated positively with COR (r = 0.468, *p* = 0.021) and GH (r = 0.484, *p* = 0.017) at POST, and CK (r = 0.492, *p* = 0.020) at POST24 when the results of SQ and LP were combined. Additionally, BL correlated positively with GH (r = 0.469, *p* = 0.021) and COR (r = 0.579, *p* = 0.003) at POST.

**Table 2:** Repeated-measures ANOVA results for maximal bilateral isometric force in isometric leg press (MIVC), maximal sEMG of quadriceps femoris over 500 ms in isometric leg press (MIVC_EMG_), countermovement jump height (CMJ), rest twitch force (RT), and serum concentrations of cortisol (COR), testosterone (TES), creatine kinase (CK), growth hormone (GH), blood lactate (BL), and blood glucose (GLUC) during Smith back squat and horizontal leg press loadings

Variable	ANOVA		
	Loading	Time	Loading × time
MIVC	*F*_(1,11)_ = 7.433	*F*_(1.8,19.5)_ = 29.024	*F*_(4,44)_ = 0.769
	*p* = 0.020	*p* < 0.001	*p* = 0.534
	ηp^2^ = 0.403	ηp^2^ = 0.725	ηp^2^ = 0.068
MIVC_EMG_	*F*_(1,11)_ = 7.202	*F*_(4,44)_ = 4.994	*F*_(4,44)_ = 5.913
	*p* = 0.021	*p* = 0.002	*p* < 0.001
	ηp^2^ = 0.396	ηp^2^ = 0.312	ηp^2^ = 0.350
CMJ	*F*_(1,9)_ = 13.116	*F*_(1.4,15.5)_ = 46.522	*F*_(2.2,24.3)_ = 0.799
	*p* = 0.004	*p* < 0.001	*p* = 0.472
	ηp_2_ = 0.544	ηp_2_ = 0.809	ηp_2_ = 0.068
RT	*F*_(1,9)_ = 13.272	*F*_(1.6,14.0)_ = 20.014	*F*_(4,36)_ = 0.842
	*p* = 0.005	*p* < 0.001	*p* = 0.508
	ηp_2_ = 0.596	ηp_2_ = 0.690	ηp_2_ = 0.086
COR	*F*_(1,11)_ = 35.988	*F*_(5,55)_ = 39.007	*F*_(2.9,31.8)_ = 8.232
	*p* < 0.001	*p* < 0.001	*p* < 0.001
	ηp^2^ = 0.766	ηp^2^ = 0.780	ηp^2^ = 0.428
TES	*F*_(1,11)_ = 5.633	*F*_(5,55)_ = 12.660	*F*_(2.3,25.1)_ = 0.947
	*p* = 0.037	*p* < 0.001	*p* = 0.412
	ηp^2^ = 0.339	ηp^2^ = 0.535	ηp^2^ = 0.079
CK	*F*_(1,10)_ = 2.311	*F*_(1.1,11.5)_ = 135.429	*F*_(1.1,10.6)_ = 3.707
	*p* = 0.159	*p* = 0.003	*p* = 0.080
	ηp^2^ = 0.188	ηp^2^ = 0.566	ηp^2^ = 0.270
GH	*F*_(1,11)_ = 8.728	*F*_(1.6,17.6)_ = 35.836	*F*_(1.3,14.0)_ = 4.680
	*p* = 0.013	*p* < 0.001	*p* = 0.041
	ηp^2^ = 0.442	ηp^2^ = 0.925	ηp^2^ = 0.298
BL	*F*_(1,11)_ = 4.568	*F*_(1.6,17.4)_ = 88.351	*F*_(1.7,18.3)_ = 4.610
	*p* = 0.056	*p* < 0.001	*p* = 0.029
	ηp^2^ = 0.293	ηp^2^ = 0.889	ηp^2^ = 0.295
GLUC	*F*_(1,11)_ = 10.042	*F*_(1.4,15.2)_ = 4.343	*F*_(3,33)_ = 8.423
	*p* = 0.009	*p* = 0.044	*p* < 0.001
	ηp^2^ = 0.477	ηp^2^ = 0.283	ηp^2^ = 0.434

### Effect of peripheral fatigue on acute neuromuscular responses

**Fig. 6:**
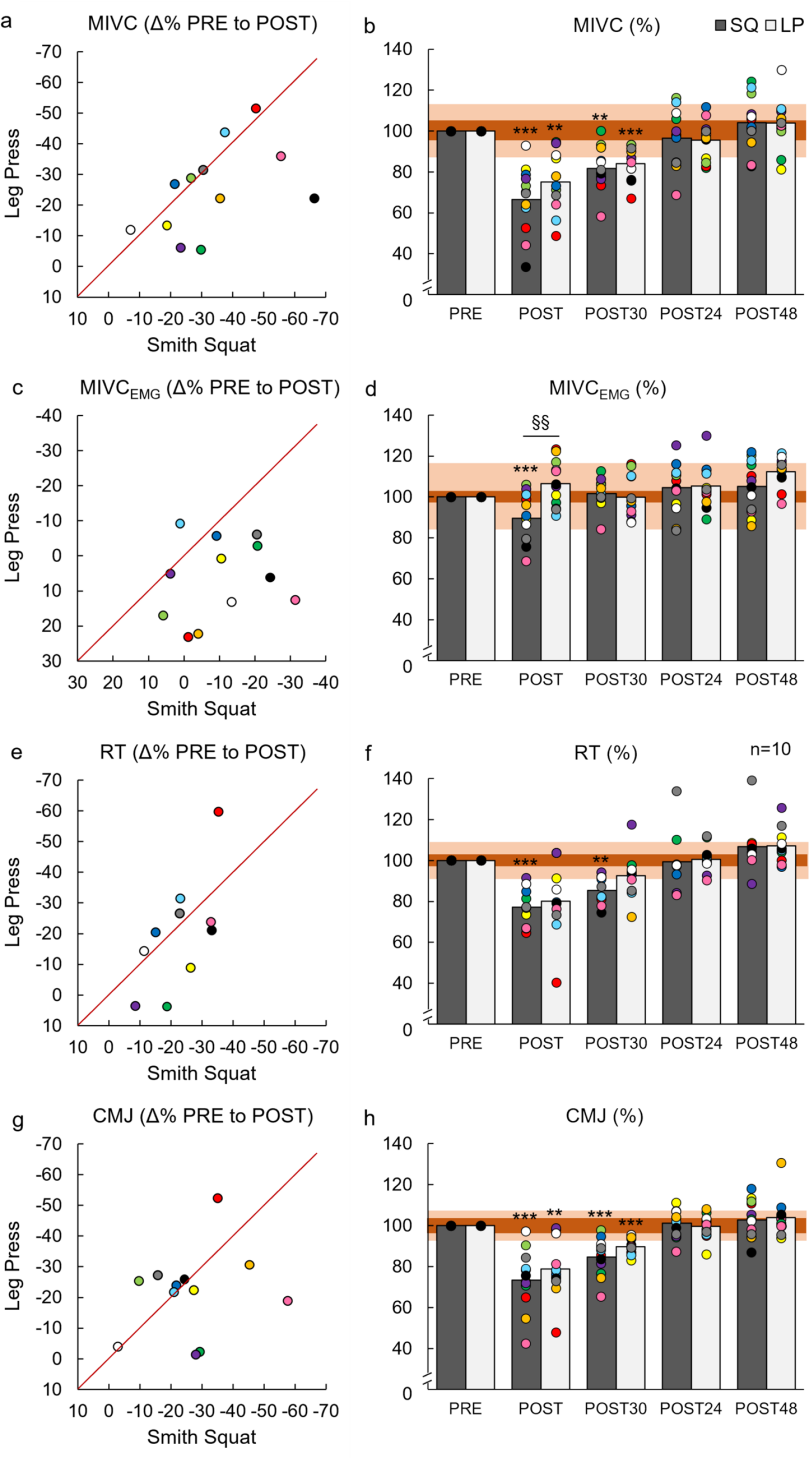
Comparison of percentage changes in maximal bilateral isometric force in isometric leg press (MIVC, **a**), maximal sEMG of quadriceps femoris muscles over 500 ms during isometric leg press (MIVC_EMG_, **c**), rest twitch force (RT, **e**), and countermovement jump height (CMJ, **g**) from before (PRE) to immediately after (POST) Smith back squat (SQ) and horizontal leg press (LP) loadings. In addition, mean and individual responses in MIVC (**b**), MIVC_EMG_ (**d**), RT (**f**), and CMJ (**h**) measured at PRE, POST, and after recovery of 30 min (POST30), 24 h (POST24) and 48 h (POST48). The light orange-shaded area represents responsiveness to loadings (i.e., change larger than two times the typical error of measurement) and the dark orange-shaded area represents the smallest worthwhile change thresholds (0.2 × between-participants standard deviation). *p < 0.05, **p < 0.01, ***p < 0.001 significant within-loading difference compared to PRE

**Fig. 7:**
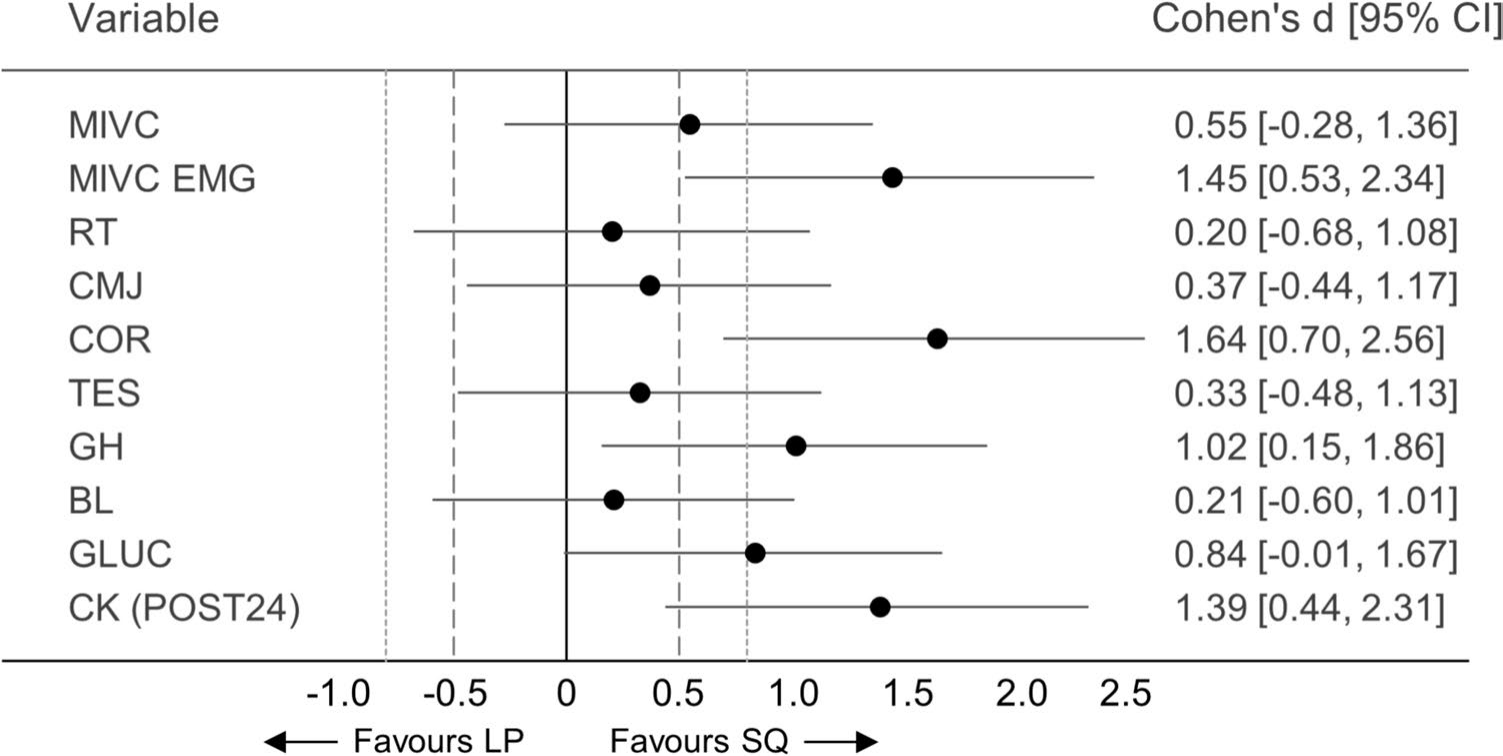
Effect size values (Cohen’s d) with 95% confidence interval (95% CI) for percentage changes from before (PRE) to immediately after (POST) Smith back squat and horizontal leg press loadings of maximal bilateral isometric force in isometric leg press (MIVC), maximal sEMG of quadriceps femoris muscles over 500 ms during isometric leg press (MIVC_EMG_), rest twitch force (RT), and countermovement jump height (CMJ). In addition, effect sizes with 95% CI for absolute serum concentrations for cortisol (COR), testosterone (TES), growth hormone (GH), lactate (BL), and glucose (GLUC) at POST and for creatine kinase (CK) after recovery of 24 h (POST24)**.** Effect size based on Cohen’s d, where small effect = 0.2, medium effect = 0.5 (long dash line), and large effect = 0.8 (dot dash line)

**Fig. 8:**
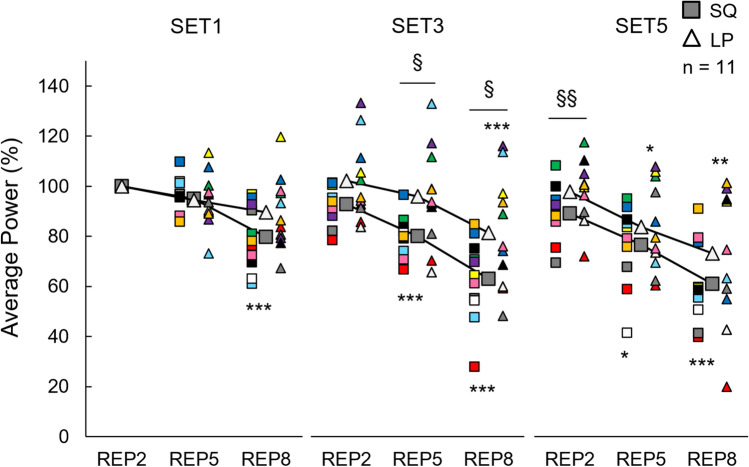
Mean and individual percentage changes in average power during the concentric phases of the selected repetitions of Smith back squat (SQ) and horizontal leg press (LP) loadings. §*p* < 0.05, §§*p* < 0.01, §§§*p* < 0.001 significantly between loadings difference, *p < 0.05, **p < 0.01,***p < 0.001 significant within-loading difference compared REP2 of same set

For neuromuscular responses, significant loading × time interaction was found for MIVC_EMG_ (*F*_(4,44)_ = 5.913, *p* < 0.001, ηp^2^ = 0.350), and pairwise comparisons indicated that a significantly greater percentage decrease in MIVC_EMG_ occurred at POST for SQ (− 10.4 vs 6.4%, *p* < 0.002, Fig. 6). No significant loading × time interactions were found for other neuromuscular variables. However, effect sizes for percentage changes from PRE to POST (Δ%) in MIVC, CMJ, and RT indicated small-to-moderate effects (*d* = 0.20–0.55, Fig. 7). In addition, significant effects for time were found for all neuromuscular variables (Table 2). A significant effect for loading was found for AP within loading sets (*p* < 0.01, ηp^2^ = 0.606), indicating a significantly greater percentage decrease in AP during SQ (*p* < 0.01, Fig. 8).

When inspecting the combined results of the loadings, strong and very strong positive correlations were found between Δ% RT from PRE to POST and Δ% MIVC (*r* = 0.821, *p* < 0.001) and Δ% CMJ (*r* = 0.768, *p* < 0.001). In addition, a positive correlation was found between Δ% RT and Δ% AP within loading sets (*r* = 0.585, *p* = 0.007). Negative correlations were found between BL POST and Δ% RT (*r* = − 0.741, *p* < 0.001), Δ% MIVC (*r* = − 0.604, *p* = 0.002), and Δ% CMJ (*r* = 0.669, *p* < 0.001). The total duration of sets correlated negatively with Δ% RT (*r* = − 0.480, *p* = 0.032) and Δ% AP within loading sets (*r* = − 0.620, *p* = 0.002).

### Recovery

COR and TES had returned to baseline before POST24, whereas CK remained elevated at this point after SQ. All measured neuromuscular variables returned to baseline before POST24.

## Discussion

The present study examined acute neuromuscular and hormonal responses, as well as recovery, during resistance exercise loadings using Smith machine SQ and horizontal LP exercises. Our primary findings indicated that exercise selection significantly influenced GH, COR, BL, and GLUC responses, with greater increases observed during SQ compared to LP, supporting our hypothesis. In contrast, exercise selection had a more limited effect on neuromuscular fatigue from PRE to POST, as only a significant difference between the loadings was observed in MIVC_EMG_, with a greater decrease occurring during SQ. Furthermore, exercise selection did not affect the recovery rate over 48 h. These limited differences in acute neuromuscular fatigue and recovery rate of neuromuscular variables contrast our hypothesis, as greater acute neuromuscular fatigue with a slower recovery rate was expected due to greater muscle activation, magnitude of activated muscle mass, and total work performed during SQ. Both neuromuscular and blood-based parameters, except creatine kinase after SQ, had returned to baseline before POST24 recovery measurements of both loadings.

### Total work and duration of sets

Significantly greater total work was performed during SQ compared to LP. This was due to the calculation of total work, which took into account vertical displacement and system mass (external mass, i.e., load and body mass). Based on a previous study (Pataky et al. 2003), it was estimated that approximately 88% of body mass is involved in SQ, excluding the mass of the leg proximal to the knee. In contrast, body mass most likely had minimal effect on work performed in LP due to the horizontal movement pattern, and thus, body mass was not included when calculating total work performed during LP. Contrary to total work, no significant differences in the total duration of sets were observed between the loadings. However, substantial inter-individual variability occurred in the total duration of sets, because the participants were instructed to perform the repetitions at their self-selected natural tempo.

### Effect of total work on blood-based parameters

Both SQ and LP loadings led to significant acute increases in COR, GH, and TES, supporting the previous research (Häkkinen and Pakarinen 1993; Smilios et al. 2003; McCaulley et al. 2009; Bartolomei et al. 2017; Kotikangas et al. 2022). Notably, acute increments in COR and GH were significantly greater during the present Smith machine SQ compared to horizontal LP, aligning with a previous study (Shaner et al. 2014) reporting greater acute hormonal responses after high-volume free-weight SQ loading compared to the inclined LP loading. In addition, we found moderate positive correlations between total work and COR and GH at POST, supporting the findings of Shaner et al. (2014). Acute increases in serum hormone concentrations, especially COR and GH, have been proposed to be dependent on the metabolic stress induced by resistance exercise (Häkkinen and Pakarinen 1993; Kang et al. 1996; Kraemer and Ratamess 2005; Shaner et al. 2014; Haunhorst et al. 2022). Our results further support this relationship, as higher BL, an approximate estimation of metabolic stress, was associated with higher COR and GH at POST. Interestingly, the present BL responses were unrelated to total work and total duration of sets, which have been proposed as important factors for metabolic stress (Gorostiaga et al. 2012; Shaner et al. 2014; Wilk et al. 2020; Corradi et al. 2021). An alternative explanation could be the difference in the magnitude of activated muscle mass between the loadings, as SQ exercise activates greater extent the posterior chain (e.g., hamstring and gluteus muscles) and trunk muscles compared to horizontal LP and requires greater muscle activity also from the synergist muscles due to the increased stabilization demands (Wilk et al. 1996; Escamilla et al. 1998; Escamilla et al. 2001; Clark et al. 2012). Taken together, these findings may suggest that larger total work and greater activated muscle mass during SQ led to greater overall physiological demand, providing a more potent stimulus for the endocrine system and resulting in larger increases in COR and GH (Tesch et al. 1986; Häkkinen and Pakarinen 1993; Kraemer and Ratamess 2005; Shaner et al. 2014; Haunhorst et al. 2022).

In contrast with Shaner et al. (2014), who reported a significantly greater acute increase in TES during free-weight SQ loading employing comparable loading protocol (6 sets at 10RM), no difference in TES response was observed between the present loadings. The degree of the increments in TES is influenced greatly by the total work performed and muscle mass engaged in the exercise (Vingren et al. 2010), so it would have been expected that the present SQ would have led to a greater increase in TES. The conflicting results between the present study and Shaner et al. (2014) may be explained first by the differences in the nutritional status of the participants (non-fasted vs. fasted) between the studies. Food intake could reduce the acute increase of TES (Hooper et al. 2017), and possibly explain more modest increases in TES during the present study, as the participants had consumed a meal 1.5–2 h before the measurements. Second, the difference in the timing of blood sampling (12–9 PM vs. 8–10 AM) could have affected the observed results, as TES follows a distinct circadian pattern with levels highest in the early morning and slowly declining over the day (Guignard et al. 1980). Thus, the acute effect of resistance exercise on TES could also differ depending on the time of day (Hooper et al. 2017). Third, the training status of the participants differed between our study and the study of Shaner et al. (2014), i.e., no systematic resistance-training experience vs. at least 6 months of resistance-training experience, respectively. In a previous study (Ahtiainen et al. 2004), resistance-trained individuals produced significantly greater acute increases in TES, possibly due to the recruitment of a larger muscle mass during resistance exercise. Thus, the differences in resistance-training experience might partly explain the inconsistent findings between the present study and the study of Shaner et al. (2014).

### Effect of peripheral fatigue on acute neuromuscular response

Substantial decrements in MIVC, CMJ, and RT from PRE to POST and AP within loading sets occurred during both SQ and LP loadings agreeing with several previous studies utilizing different types of SQ and LP exercises (e.g., Häkkinen and Pakarinen 1993; McCaulley et al. 2009; Walker et al. 2012; Peltonen et al. 2013; Kotikangas et al. 2022). High-volume resistance exercise loadings have been reported to cause various biochemical changes at the muscle cell level (Gorostiaga et al. 2012). These include reductions in ATP and PCr levels and the capacity to regenerate ATP (Tesch et al. 1986; Gorostiaga et al. 2012), with simultaneous elevations in inorganic phosphate and ion concentrations (e.g., Mg^2+^) impacting myofibrillar force production, Ca^2+^ sensitivity, and Ca^2+^ release from the sarcoplasmic retinaculum (Allen et al. 2008; Cheng et al. 2018). Additionally, disturbance in intra- and extracellular Na^+^ and K^+^ concentrations could further lead to decreased muscle excitability (Cheng et al. 2018), impacting force and power production. The significantly reduced RT during both loadings indicated impaired muscle contractility, possibly due to these biochemical changes.

The decrements in neuromuscular variables were most likely related to the aforementioned biochemical changes at the peripheral level, since percentage decreases in RT were strongly associated with percentage decreases in MIVC and CMJ from PRE to POST and within-set decrements in AP. The present findings also indicate that the inter-individual variation in the magnitude of acute neuromuscular fatigue seemed to be explained by the magnitude of peripheral fatigue. In the previous studies, researchers have demonstrated that a longer time under tension can lead to greater decreases in muscle contractile properties and power production (Tran et al. 2006; Corradi et al. 2021). In agreement with the previous findings (Tran et al. 2006; Corradi et al. 2021), the longer duration of sets (i.e., time under tension) was associated with greater percentage decreases in RT from PRE to POST. Thus, longer total duration of sets likely led to more pronounced peripheral fatigue, for instance, due to more substantial disruption of ion concentrations and greater depletion of ATP and PCr (Tran et al. 2006; Gorostiaga et al. 2012; Thomas et al. 2018; Wilk et al. 2020; Corradi et al. 2021), and this was noted as greater neuromuscular performance decrements at the individual level.

Despite significantly greater total work and within-set decrements in AP during the present SQ, neuromuscular fatigue progressed similarly from PRE to POST across both loadings, except for the greater decrease in MIVC_EMG_ observed during SQ. One possible explanation for minimal differences in acute neuromuscular responses could be that the smaller magnitude of activated muscle mass during the present LP allowed the development of greater local peripheral fatigue before considerable disruptions occurred in physiological homeostasis and fatigue sensation became intolerable (Thomas et al. 2018). Our findings partly supported this explanation, as half of the participants demonstrated larger decrements in RT from PRE to POST during LP. In addition, a more pronounced disturbance of physiological systems during fatiguing exercises engaging larger muscle mass (e.g., during the present SQ) may be intended to be limited by restraining central motor command via heightened inhibitory feedback from group III/IV afferents (Thomas et al. 2018). Significantly higher BL at MID during SQ could give an estimation of greater physiological demand, and a greater decrease in MIVC_EMG_ may provide indirect evidence of the possibility of heightened central inhibition during SQ. Additionally, more rapid disruption in physiological systems with the increased central inhibition may explain why the barbell load had to be decreased substantially set by set after SET3 of SQ for the participants to be able to continue work (Amann et al. 2013; Rossman et al. 2014). For this reason, the training stimulus might have been reduced more at the latter part of SQ compared to LP, where only minimal decreases in the load were required. Thus, the maintained work output throughout all sets of LP, together with greater local peripheral fatigue in QF muscles, could explain similar neuromuscular fatigue responses observed after the completion of the loadings.

### Recovery after the loadings

The recovery progressed similarly after both loadings, which was expected given the similar magnitude of acute neuromuscular fatigue between the loadings. All neuromuscular variables, COR and TES had returned to the baseline levels before POST24 measurements, suggesting that 24 h is a sufficient time period for the neuromuscular and hormonal systems to recover from the present high-volume resistance exercises. This finding may imply that these types of loadings are reasonable training stimuli even for novices as other researchers have reported reduced neuromuscular performance and TES even after 48 h of recovery after very high-volume resistance exercise loadings (Häkkinen and Pakarinen 1993; Bartolomei et al. 2017). Another interesting finding was that CK increased significantly only after SQ at POST24 and POST48 indicating possibly heightened exercise-induced muscle damage after SQ (Bartolomei et al. 2017). Moreover, CK seemed to be related to the magnitude of total work (*r* = 0.492, *p* = 0.020), suggesting that SQ could have led to a heightened degree of exercise-induced muscle damage compared to LP due to the greater magnitude of total work performed during SQ.

It should be acknowledged that considerable differences in the recovery rate were observed between the participants. The recovery rate after resistance exercises could be influenced by various factors, including training intensity, volume, the degree of acute fatigue and metabolic stress induced by the exercise, training experience, strength levels of the individual, nutrition, sleep, and genetics (Bartolomei et al. 2017; Kraemer et al. 2017; Ishida et al. 2023). Based on our results, the recovery rate of individuals at POST24 could be partly related to the magnitude of muscle damage, since higher CK was associated with larger decrements in CMJ (*r* = − 0.458, *p* = 0.032) and RT (*r* = − 0.686, *p* = 0.003) at POST24. In addition, the degree of acute fatigue could be one possible explanation, because greater fatigue in MIVC from PRE to POST was associated with greater decrements in CMJ at POST24 (*r* = 0.484, *p* = 0.017). Stronger individuals, consequently performing higher total work, also demonstrated a slower recovery rate during LP, since CMJ at POST24 was negatively correlated with 1RM in LP (*r* = − 0.792, *p* = 0.002) and total work (*r* =− 0.724, *p* = 0.008). Thus, it seems that the recovery rate after the present loadings was affected by a combination of factors.

### Limitations

There were some limitations that need to be acknowledged. First, the absence of kinematic data is a notable limitation, as it would have provided a more comprehensive understanding of joint angles, movement patterns, and potential differences in execution between Smith machine SQ and horizontal LP exercises, thereby strengthening the interpretation of the present findings. Second, the order of loading sessions was not randomized due to scheduling reasons within the larger research project. This lack of randomization may have influenced the results due to the repeated bout effect, i.e., a phenomenon where the body adapts to a novel training stimulus, and the acute responses to the second similar training stimulus are considerably smaller (McHugh 2003). However, to minimize this effect, loading sessions were separated by at least 7 days (mean 32 days). In addition, potential learning effects in neuromuscular testing after the first session may have contributed to improved performance during the second loading week (Chrzanowski-Smith et al. 2020). This learning effect was intended to be minimized by organizing the familiarization session, where the participants were allowed to practice all test procedures multiple times. Notably, this limitation had minimal impact on the results, as no significant differences in PRE values of SQ and LP were observed. Finally, variation in the timing of loading sessions (ranging from noon and 9 pm) may have contributed to variability in acute responses between participants.

### Conclusions

Our results suggest that Smith machine SQ may provide a more potent stimulus for metabolic and hormonal responses during high-volume resistance exercise loading compared to horizontal LP, at least partly due to the greater total work performed. However, horizontal LP should not be overlooked as a valuable exercise for targeting the quadriceps femoris muscles, particularly in rehabilitation and hypertrophy-focused training, as it appears to fatigue quadriceps femoris muscle in a similar manner as the Smith machine SQ. Overall, exercise selection had a more limited effect on the acute neuromuscular responses from PRE to POST and over a 48 h recovery period. The observed decrements in neuromuscular variables appeared to be mostly related to peripheral factors affecting muscle contractile properties of quadriceps femoris muscle, although central components cannot be entirely ruled out based on the present methods. Furthermore, the total duration of sets, i.e., time under tension, was linked to the magnitude of peripheral fatigue, explaining inter-individual variability in acute neuromuscular responses during both loadings. Future research should focus on conducting a full biomechanical analysis of SQ and LP loadings (including, e.g., muscle activation and joint angles and torques) and examining how the progression of neuromuscular fatigue affects these biomechanical aspects during the loading itself.

## Data Availability

All data are available from the corresponding author upon reasonable request.

## Abbreviations

1RM/3RM/10RM: One/three/ten-repetition maximum
ANOVA: Repeated-measures analysis of variance
BL: Blood lactate
CK: Creatine kinase
CMJ: Countermovement jump
COR: Cortisol
GH: Growth hormone
GLUC: Blood glucose
LP: Horizontal leg press
MID: Measurement point after third loading set
MIVC: Maximal bilateral isometric force in isometric leg press
MIVC_EMG_: Maximal sEMG of quadriceps femoris over 500 ms period in isometric leg press
PRE: Measurement point before loading protocol
POST: Measurement point immediately after loading protocol
POST30/POST24/POST48: Measurement points after 30 min, or 24/48 h of recovery
QF: Quadriceps femoris
RF: Rectus femoris
RT: Potentiated rest twitch force during electrical stimulation
sEMG: Surface electromyography
SQ: Back squat
SWC: Smallest worthwhile change
TE: Typical error of measurement
TES: Testosterone
VL: Vastus lateralis
VM: Vastus medialis
